# Retracted Randomized Clinical Trials From Superretractors and Top-Cited Scientists With Multiple Retractions

**DOI:** 10.1001/jamanetworkopen.2026.7424

**Published:** 2026-04-15

**Authors:** Chunwei Lyu, Minoo Matbouriahi, Florian Naudet, John P. A. Ioannidis, Ioana Alina Cristea

**Affiliations:** 1 Department of General Psychology, University of Padova, Padova, Italy; 2 University Rennes, CHU Rennes, Inserm, EHESP, Irset (Institut de recherche en santé, environnement et travail)–UMR_S 1085, Centre d'investigation clinique de Rennes (CIC1414), Rennes, France; 3 Institut Universitaire de France (IUF), Paris, France; 4 Meta-Research Innovation Center at Stanford (METRICS), Stanford University, Stanford, California; 5 Stanford Prevention Research Center, Department of Medicine, Stanford University School of Medicine, Stanford, California; 6 Department of Epidemiology & Population Health, Stanford University School of Medicine, Stanford, California; 7 Department of Biomedical Data Science, Stanford University School of Medicine, Stanford, California

## Abstract

**Importance:**

Multiple retractions from the same author often uncover issues affecting their entire work, such as having systematically altered or fabricated data.

**Objectives:**

To evaluate the contribution of authors with the most retractions (ie, superretractors) and top-cited scientists with multiple retractions to the retracted randomized clinical trial (RCT) literature.

**Design, Setting, and Participants:**

This retrospective cohort study linked an openly available cohort of retracted RCTs (VITALITY) to 3 lists of scientists: (1) superretractors, totaling most retractions in the Retraction Watch Leaderboard; (2) scientists in the top 100 000 or 2% of their subfield in terms of citations (ie, top-cited scientists) over their entire careers who accumulated 10 or more retractions not due to editor or publisher errors; and (3) top-cited scientists in the most recent year (ie, 2024) who accumulated 10 or more retractions not due to editor or publisher errors. The VITALITY cohort was updated up to November 2024. The 3 author lists were updated in August 2025.

**Main Outcomes and Measures:**

The main outcomes were authorship and the characteristics of retracted RCTs (publication and retraction year, time between publication and retraction, number of citations).

**Results:**

A total of 30 superretractors, 163 career-long top-cited scientists with 10 or more retractions, and 174 recent-year top-cited scientists with 10 or more retractions were included; 1330 retracted RCTs were included. Overall, 6 superretractors (20%), representing anesthesiology as well as endocrinology and metabolism, coauthored 290 retracted RCTs (22%); 18 career-long top-cited scientists with at least 10 retractions, representing 10 fields, coauthored 327 trials (25%), 275 (84%) of which were also coauthored by a superretractor; 7 single-year top-cited scientists with at least 10 retractions coauthored 50 retracted RCTs (4%), all of which were also included in the list of articles authored by career-long top-cited scientists with at least 10 retractions. Articles with superretractor authors vs not were published earlier (median [IQR], 2000 [1997-2005] vs 2020 [2014-2022]); retracted earlier (median [IQR], 2013 [2012-2019] vs 2023 [2018.5-2023]); had a longer lag between publication and retraction (median [IQR], 5111 [3560-6820] days vs 482 [330-1119] days); and accrued more citations (median [IQR], 21 [12-42] vs 5 [1-19]). In multivariable regression models, only time to retraction (β = 0.02; *P* < .001) was significantly and positively associated with total citations. Results were similar when comparing retracted articles from top-cited scientists with at least 10 retractions vs other articles.

**Conclusions and Relevance:**

In this cohort study of 1330 retracted RCTs, a small number of influential authors, often coauthors and concentrated across few fields of medicine, accounted for a significant proportion of retracted clinical trials.

## Key Points

**Question:**

How many retractions have authors with the most retractions (ie, superretractors) and top-cited authors with multiple retractions contributed to the retracted randomized clinical trial literature?

**Findings:**

In this cohort study of 367 authors and 1330 retracted randomized clinical trials, 6 superretractors, from anesthesiology as well as endocrinology and metabolism, coauthored one-fifth of all retracted trials, while 18 top-cited scientists with more than 10 retractions coauthored one-quarter of them. Articles coauthored by superretractors or by top-cited scientists with multiple retractions were published and retracted earlier, took longer to retract, and accumulated more citations.

**Meaning:**

This study found that retracted randomized clinical trials were disproportionately associated with a small number of influential authors, often coauthors, and concentrated across few subfields of medicine.

## Teaser

This cohort study evaluates the author and publication characteristics of retracted randomized clinical trials.

## Introduction

Retracted randomized clinical trials (RCTs) have an enduring and multiplicative impact on the evidence ecosystem by distorting findings of systematic reviews and meta-analyses,^1,2^ which then percolate into clinical practice guidelines.^1^ Concerningly, only a part of RCTs that would qualify for a retraction end up being retracted.^3^ According to the guidelines by the Committee on Publication Ethics (COPE), “clear evidence of major irregularities in the data or images…that compromise the reliability of the findings”^4^ would warrant retraction. By this metric, so-called zombie RCTs,^5^ ie, RCTs that appear to be fake or where data blatantly lack credibility, should all have been either rejected by journals or retracted.^6^ This does not seem to be happening. For example, employing the Investigating Problematic Clinical Trials in Systematic Reviews (INSPECT-SR) tool for assessing the trustworthiness of 95 RCTs included in Cochrane systematic reviews, 24 (25%) and 6 (6%) were identified as having some and serious concerns, respectively.^7^ Only 2 of these flagged trials had been retracted or accompanied by expressions of concern.

Previous research has described superretractors, ie, scientists who have amassed numerous retractions.^8,9^ Similarly, recent work examined influential, highly cited scientists with retractions not due to publisher or journal error,^10^ a subset of whom had more than 10 retractions. Multiple retractions from the same author often stimulate investigations that uncover issues affecting an author’s entire work, such as having systematically altered or fabricated data. However, there are no estimations of the contribution of superretractors or top-cited authors with multiple retractions to the corpus of retracted RCTs. We aimed to quantify this contribution, based on a previously published and openly available complete cohort of retracted RCTs (VITALITY).^1^

## Methods

### Ethical Approval

Per the Common Rule, ethical approval was not requested for this cohort study because it involved data extracted from published research, and the publicly available datasets on which this research was based. No human participants were involved. We followed the Strengthening the Reporting of Observational Studies in Epidemiology (STROBE) reporting guideline for cohort studies.^11^

### Study Population

The study is a retrospective cohort study. The VITALITY cohort^1^ includes 1330 RCTs with notification of retraction, identified from Retraction Watch (RW) (updated November 5, 2024). To assemble the VITALITY cohort, records labeled *clinical study* or *research articles* limited to subjects of (*HSC*) *health sciences* were retrieved from RW. Subsequently, 2 independent researchers reviewed titles, abstracts, and full-texts and selected all studies that reported the design as an RCT in the methods. Multiple analyses from the same primary RCT were retained only if data were analyzed as per original randomization. No restrictions were placed for reasons for retraction or other characteristics. References and study characteristics for the cohort are openly available.^12^ We linked the VITALITY cohort with 3 groups of authors with multiple retractions.

Group 1 came from the RW Leaderboard, August 26, 2025, update.^13^ It includes 30 researchers with the highest number of retractions (range, 33 to 221).

The 2 other author groups came from the science-wide author databases of standardized citation indicators, August 2025 update^14^: scientists who are top-cited over their career (career-long, group 2) and in 2024 (single-year, group 3). Top-cited was defined^15^ as being among the top 100 000 scientists or in the top 2% among authors in the same subfield by a composite of standardized values of citation indicators.^16^ In the science-wide author databases, retractions are only counted if not due to publisher or journal error and if the article was not replaced by a revised version. We selected scientists from the top-cited career-long and single-year cohorts who amassed at least 10 such retractions (163 scientists in group 2 and 174 in group 3).

### Data Extraction

References in the publicly available VITALITY dataset include truncated authors lists, so 1 author (C.L.) extracted the full reference that included full names of all authors for each retracted RCT, using the available digital object identifier (DOI). Subsequently, for each complete reference, 2 researchers (C.L. and M.M.) independently checked each of the authors names against the lists of scientists in groups 1, 2 and 3 using the search function in Excel for Mac version 16.86 and version 16.x (Microsoft Corp) search function and manually examining each hit. For each article, the researchers noted whether any authors from groups 1, 2, or 3 were involved as authors.

From the VITALITY dataset, we compiled characteristics of retracted trials, namely publication and retraction dates (month-date-year format), from which we calculated the time (in days) from publication to retraction. Additionally, 1 researcher (C.L.) independently extracted number of citations from Scopus as of November 21, 2025.

### Statistical Analysis

Interrater agreement was assessed with the κ statistic, separately for the 3 groups of authors (superretractors, career-long top-cited, and single-year top-cited). Results are presented descriptively as counts, proportions, and confidence intervals (CIs) calculated with the binomial exact method, and medians and IQRs. As we expected a high level of correlation between the variables considered, we conducted Pearson correlation with *P* values corrected for multiple comparisons with the Sidak correction. We compared publication and retraction year, time from publication to retraction and total citations between groups (ie, involving superretractors, top-cited career-long scientists, and top-cited single-year scientists vs not) with the Wilcoxon rank-sum test. To disentangle the effects on citation counts of authorship from superretractors or top-cited scientists with multiple retractions from survival effects (ie, articles that survive longer in the literature are cited more), we conducted multivariable linear regression analysis including presence of superretractor or top-cited author as binary variable, time between retraction and publication (in days) as continuous, and their interaction. Significant interaction effects were followed up by univariate regression separate for articles involving superretractors and top-cited scientists or not.

Analyses were conducted in Stata SE version 19.0 (StataCorp) and Claude Sonnet version 4.5 (Anthropic) was used for checking code and for data visualization. Statistical significance was set at α = .05, and all tests were 2-tailed.

## Results

There were a total of 30 superretractors, 163 top-cited career-long scientists, and 174 top-cited recent-year scientists. Overall, 15 scientists in group 2 (9%) and 8 in group 3 (5%) were also superretractors (group 1). Interrater agreement was 100% for scientists in group 1 and group 2, and 99.92% (1 discrepancy) for group 3.

Of 30 super-retractors, 6 (20%) had coauthored 290 of the 1330 retracted RCTs (22%) (Table 1), 52 of which (18%) had been coauthored by more than 1 superretractor. The 6 superretractors belonged to 2 subfields: anesthesiology (3 superretractors) and endocrinology and metabolism (3 superretractors), and all but 1 were affiliated with Japanese institutions. Retracted RCTs represented between 3% and 79% of all their retracted articles. For 3 superretractors, more than half of their retracted articles were RCTs.

**Table 1.** Retracted RCTs Authored by Known Superretractors

Superretractor	Subfield	Country	RCTs, No.^a^		Total retracted, No.^b^		Retracted RCTs vs articles (95% CI), %
			As first author	As co-author	RCTs	Articles	
Joachim Boldt	Anesthesiology	Germany	72	47	119	221	54 (47-61)
Yoshitaka Fujii	Anesthesiology	Japan	111	10	121	172	70 (63-77)
Yoshihiro Sato	Endocrinology and metabolism	Japan	26	4	30	124	24 (17-33)
Hironobu Ueshima	Anesthesiology	Japan	3	1	4	124	3 (1-8)
Jun Iwamoto	Endocrinology and metabolism	Japan	5	19	24	91	26 (18-37)
Yuhji Saitoh	Anesthesiology	Japan	20	24	44	56	79 (66-88)

^a^ According to the updated science-wide author databases of standardized citation indexes.^14^

^b^ According to the Retraction Watch Database^13^; list updated August 25, 2025.

A total of 18 of the 163 career-long top-cited scientists with at least 10 retractions (11%) coauthored 327 of 1330 retracted trials (25%) (eTable 1 in Supplement 1). Five of these authors (28%) were also superretractors. The 18 top-cited scientists covered 10 subfields, but many concentrated in anesthesiology (4 authors) and cardiovascular system and hematology (4 authors). They represented 8 countries, with most in Japan (7 authors) and the US (4 authors). Overall, 7 of 18 (39%) had coauthored only a single retracted trial, while for another 7 (39%), retracted RCTs represented half of their retracted articles. Overall, 275 of 327 retracted RCTs (84%) involved a superretractor.

A total of 7 of 174 top-cited scientists in 2024 with at least 10 retractions (4%) coauthored 50 of 1330 retracted trials (4%) (eTable 2 in Supplement 1). All 7 authors were also in the top-cited career-long scientist group. Three authors (43%) had coauthored a single retracted trial, while for 4 authors (57%), retracted RCTs represented more than half of their retracted articles. None of the authors themselves were on the RW leaderboard, but 14 of 50 (28%) retracted RCTs were coauthored with a superretractor (Yoshihiro Sato).

As displayed in Table 2, retracted RCT articles with superretractor authors, compared with those without superretractor authors, were published earlier (median [IQR], 2000 [1997-2005] vs 2020 [2014-2022]; *P* < .001), were retracted earlier (median [IQR], 2013 [2012-2019] vs 2023 [2018.5-2023]; *P* < .001), had a longer lag between publication and retraction (median [IQR], 5111 [3560-6820] days vs 482 [330-1119] days; *P* < .001); and amassed more citations (median [IQR], 21 [12-42] vs 5 [1-19]; *P* < .001). Similarly, retracted articles published by top-cited career-long scientists with at least 10 retractions, compared with other retracted RCTs, were published earlier (median [IQR], 2001 [1998-2007] vs 2021 [2014-2022]; *P* < .001), were retracted earlier (median [IQR], 2014 [2012-2020] vs 2023 [2019-2023]; *P* < .001). had a longer lag between retraction and publication (median [IQR], 4748 [2708-5872] days vs 469 [325-1058] days; *P* < .001); and had more citations (median [IQR], 24 [14-47] vs 4 [1-16]; *P* < .001). Results were similar for articles published by single-year top-cited scientists vs other retracted RCT articles (Table 2). The results of correlation analyses are displayed in eTable 3 in Supplement 1. Total citations had small, but significant negative correlations with publication year (Pearson *r* = −0.144; *P* < .001) and retraction year (Pearson *r* = −0.128; *P* < .001), and a similarly small, but significant, positive correlation with time between publication and retraction was observed (Pearson *r* = 0.097; *P* = .009). Correlations with the presence of authors from any of the 3 groups were not statistically significant.

**Table 2.** Characteristics of Retracted Randomized Clinical Trials Involving Superretractors, Career-Long and 2024 Top-Cited Scientists With at Least 10 Retractions vs Randomized Clinical Trials Not Involving Them

Group	Article, No. (%) (N = 1330)	Publication year, median (IQR)	*P* value^a^	Retraction year, median (IQR)	*P* value	Time to retraction, median (IQR), d	*P* value	Citations, median (IQR)^b^	*P* value
Superretractor									
Yes	290 (22)	2000 (1997-2005)	<.001	2013 (2012-2019)	<.001	5111 (3560-6820)	<.001	21 (12-42)	<.001
No	1040 (78)	2020 (2014-2022)		2023 (2018.5-2023)		482 (330-1119)		5 (1-19)	
Top-cited career-long scientist with ≥10 retractions									
Yes	327 (25)	2001 (1998-2007)	<.001	2014 (2012-2020)	<.001	4748 (2708-5872)	<.001	24 (14-47)	<.001
No	1003 (75)	2021 (2014-2022)		2023 (2019-2023)		469 (325-1058)		4 (1-16)	
Top-cited 2024 scientist with ≥10 retractions									
Yes	50 (4)	2010 (2005-2015)	<.001	2017 (2016-2022)	.049	2557.5 (1597-4718)	<.001	38 (18-65)	<.001
No	1280 (96)	2018 (2008-2022)		2022 (2015-2023)		611 (358-2504)		7 (2-24)	
Total	1330 (100)	2017 (2007-2022)	NA	2022 (2015-2023)	NA	637.5 (362-2690)	NA	13 (4-42)	NA

Abbreviation: NA, not applicable.

^a^ Calculated with the Wilcoxon rank-sum test.

^b^ Citation counts were extracted from Scopus. For 12 trials, number of citations was not available.

In multivariable regression models (eTable 4, eResults, and eFigure 1 in Supplement 1), time to retraction (β = 0.02; *P* < .001) but not presence of superretractors (β = 24.7; *P* = .10) was significantly and positively associated with total citations. The interaction between time to retraction and presence of superretractors was significant and negative (β = −0.02; *P* < .001). Similarly (eTable 4, eResults, and eFigure 2 in Supplement 1), both time to retraction (β = 0.01; *P* < .001) and presence of top-cited career-long scientists with at least 10 retractions (β = 35.26; *P* = .007) were significantly and positively associated with number of citations, but their interaction had a significant and negative association (β = −0.01; *P* < .001). For articles coauthored by top-cited scientists in 2024 with at least 10 retractions (eTable 4 in Supplement 1), only time to retraction was positively associated with citation counts (β = 0.004; *P* = .001), and there was no significant interaction effect.

## Discussion

In this study, 6 superretractors, representing 2 fields of medicine, accounted for one-fifth of all retracted RCTs. Similarly, 18 career-long top-cited scientists with more than 10 retractions accounted for a quarter of all retracted RCTs. All but 1 of the superretractors involved in retracted RCTs were also among the top-cited scientists over their career. There was a large overlap between retracted trials attributed to superretractors and those attributed to career-long highly cited scientists who amassed at least 10 retractions. Retracted RCT articles coauthored by superretractors or by top-cited scientists with multiple retractions were published and retracted earlier, took longer to retract and, in uncorrected analysis, accumulated more citations than retracted RCT articles not involving such authors. In correlation analysis, retraction and publication year, as well as time to retraction, showed modest associations with citation counts, but not author status. In multivariable analysis, time to retraction was consistently positively associated with citations, as expected, while author status was no longer significantly associated with citations. There was a significant interaction effect between time to retraction and author status: for articles by authors who were not superretractors or top-cited over their career, citations gradually increased over time, while there was no such trend for articles attributed to superretractors or top-cited career-long scientists.

Multiple retractions from the same author stimulate efforts to inspect other publications belonging to them, often leading to more retractions. For example, the work of superretractors has been thoroughly scrutinized, with an analysis^17^ from 2019 indicating that only 9% of the articles eligible for retraction remained not retracted. Conversely, the work of top-cited scientists with many retracted publications has received less attention; additional articles by these authors may deserve scrutiny. Nevertheless, the enhanced examination likely led to a substantial overrepresentation of publications from superretractors or top-cited scientists with multiple retractions within the current population of retracted RCTs. As probably all articles from superretractors were examined and many subsequently retracted, the cohort of retracted trials included a mix of more consequential, and thus more cited, articles as well as more peripheral ones, which received significantly less attention from the scientific community. A similar phenomenon could have occurred to articles from top-cited career-long scientists, which largely overlapped with articles attributed to superretractors. Additional scrutiny to top-cited scientists who had fewer than 10 retractions, so were not included in our analysis, might lead to future retractions and increase their representation within the cohort of retracted trials. Superretractor articles amassed more citations probably mostly because they had more time before they were retracted. However, some of the authors with retractions who are currently not superretractors may eventually have additional retractions and may reach superretractor status in the future. By then, it is possible that their late-retracted articles will have accumulated many more citations before being taken off the literature. In contrast, articles from researchers top cited in 2024 were fewer, and only time to retraction was associated with citation counts. Some of the authors who currently have few, sporadic retractions may end up accumulating more retractions in the future, and they may also enter the group of superretractors.

Retracted RCTs belonging to superretractors or scientists top cited over their entire career were published earlier and took longer to retract, meaning there was more time to identify and investigate publications, formulate allegations, and follow up on retractions. For example, concerns about Yoshihiro Sato,^9^ one of the most prolific superretractors, had been raised almost 10 years before the first retractions occurred, in letters to the editor, and systematic investigation of his work by other researchers took almost 3 years to get published^18^ and for the first retractions to begin. Moreover, investigation of multiple articles from the same author often attracts examination of coauthors,^9^ which in the case of Sato, led to another superretractor in his frequent coauthor, Jun Iwamoto. Another frequent coauthor, Kei Satoh, was only included on the top-cited career-long list in the current analysis.^9^ Coauthorship patterns at least partly account for the overrepresentation of some countries in our cohort of retracted RCTs.

Beyond investigation of coauthors of prolific retractors, some fields, most notably anesthesia, have been extensively scrutinized, also leading to overrepresentation in our sample. An analysis^19^ of anesthesia articles retracted until 2017 identified 350 retracted articles, with 4 individuals responsible to nearly 60% of all retracted articles. Although these articles were not restricted to RCTs, 2 of the 4 authors identified (Yoshitaka Fujii and Joachim Boldt) were also on our list of superretractors and top-cited career-long authors of RCTs and another (Scott S. Reuben) only on the top-cited list.^17^ These authors worked independently on different topics, so their large, combined output of retracted RCTs cannot be attributed to coauthorship.

Notably, several authors with many retractions were top cited for their career-long citation impact, but only a minority of them continued to be top cited in the most recent single year. This may reflect reputational loss and decline in citations to their publication corpus. An earlier analysis^20^ evidenced that authors who had a retraction experienced a citation penalty of 10% (20% if the retraction was related to misconduct) for their nonretracted work. This penalty is likely more severe for authors with multiple retractions and might extend to their frequent coauthors. Concentration of prolific retractors in some subfields and countries may reflect either systemic problems in these subfields and countries or better sensitization toward detecting fraud. Conversely, it is possible that the overrepresentation of some fields and countries masks insufficient scrutiny of other fields or inadequate detection methods in other countries.

### Limitations

Our study has several limitations. First, retractions represent only a fraction of the literature that should be retracted, following guidelines such as COPE.^4^ Retracted RCTs, even when clearly linked to data fabrication, fraud, or severe methodological problems, are not randomly selected sample of zombie trials,^5^ which limits drawing inferences about factors associated with misconduct or inadequate research practices from a cohort of retracted trials. Some authors with few retracted articles may accumulate more retractions in the future, and some fields are receiving heightened scrutiny, such as obstetrics and gynecology,^21,22^ potentially changing the profiles emerging in our current data. Recent proposals^22^ to examine the collected body of evidence coming from an author or author group suspected of misconduct might uncover other superretractors. Second, the VITALITY cohort includes trials retracted for any reason, so also for problems not related to data fabrication or severe flaws, thus precluding the use of retraction as a proxy for misconduct or fraud. However, all 3 lists of authors included researchers who accumulated multiple retractions for reasons not imputable to publisher or journal error, thereby increasing the likelihood that their accumulated retractions reflect at least inadequate research practices, if not overt misconduct. Third, analysis of citations is confounded by the fact retracted articles continue to be cited after retraction,^23^ even when retraction was explicitly linked to misconduct,^24^ and we did not distinguish citations before or after the retraction. Fourth, the dates of update of the 3 cohorts included are slightly different, with VITALITY dataset updated in November 2024 and the 3 author groups in August 2025. Moreover, citation counts for VITALITY cohort were extracted de novo in November 2025, although this update is unlikely to have produced a major shift in citations.

## Conclusions

In this cohort study, retracted RCTs were associated with a small number of authors—often coauthors—and concentrated across a few medical subfields and countries. The disproportionate role of superretractors and top-cited authors with multiple retractions, their interconnections as expressed by coauthorships, and the narrow concentration may provide useful leads to help unravel fraudulent literature at scale.

## Notes

Supplement 1. **eTable 1.** Retracted Randomized Trials Authored by Career-Long Top-Cited Scientists With at Least 10 Retractions **eTable 2.** Retracted Randomized Trials Authored by 2024 Top-Cited Scientists With at Least 10 Retractions **eTable 3.** Correlation Between the Main Variables Extracted **eTable 4.** Multivariable Linear Regression Analysis With Total Citations (Scopus) as Dependent Variable
eResults.
**eFigure 1.** Mean Citations by Survival Time and Superretractor Status in 200-Day Bins **eFigure 2.** Mean Citations by Survival Time and Top-Cited Career-Long Status in 200-Day Bins

Supplement 2.
Data Sharing Statement
