# Supplementary material for: Retracted Randomized Clinical Trials From Superretractors and Top-Cited Scientists With Multiple Retractions
Source: JAMA Netw Open. 2026 Apr 15;9(4):e267424. doi: 10.1001/jamanetworkopen.2026.7424 (PMC13084457; doi:10.1001/jamanetworkopen.2026.7424)
Supplement: Supplement 1. — eTable 1. Retracted Randomized Trials Authored by Career-Long Top-Cited Scientists With at Least 10 Retractions eTable 2. Retracted Randomized Trials Authored by 2024 Top-Cited Scientists With at Least 10 Retractions eTable 3. Correlation Between the Main Variables Extracted eTable 4. Multivariable Linear Regression Analysis With Total Citations (Scopus) as Dependent Variable eResults. eFigure 1. Mean Citations by Survival Time and Superretractor Status in 200-Day Bins eFigure 2. Mean Citations by Survival Time and Top-Cited Career-Long Status in 200-Day Bins [file jamanetwopen-e267424-s001.pdf]

Supplemental Online Content

Lyu C, Matbouriahi M, Naudet F, Ioannidis JA, Cristea IA. Retracted randomized clinical trials from superretractors and top-cited scientists with multiple retractions. *JAMA Netw Open.* 2026;9(4):e267424. doi:10.1001/jamanetworkopen.2026.7424

eTable 1. Retracted Randomized Trials Authored by Career-Long Top-Cited Scientists With at Least 10 Retractions

eTable 2. Retracted Randomized Trials Authored by 2024 Top-Cited Scientists With at Least 10 Retractions

eTable 3. Correlation Between the Main Variables Extracted

eTable 4. Multivariable Linear Regression Analysis With Total Citations (Scopus) as Dependent Variable

eResults.

eFigure 1. Mean Citations by Survival Time and Superretractor Status in 200-Day Bins

eFigure 2. Mean Citations by Survival Time and Top-Cited Career-Long Status in 200-Day Bins

This supplemental material has been provided by the authors to give readers additional information about their work.

© 2026 Lyu C et al. *JAMA Network Open*.

eTable 1. Retracted Randomized Trials Authored by Career-Long Top-Cited Scientists With at Least 10 Retractions

Top-cited (career-long) & ≥ 10 retractions	Subfield[a]	Country	RCTs as 1st author	RCTs as co-author	Total retracted RCTs	Total retracted articles[a]	% retracted RCTs vs articles (95% CI)
Yoshitaka Fujii[b]	Anesthesiology	JP	111	10	121	168	72 (65 – 79)
Joachim Boldt[b]	Anesthesiology	DE	72	47	119	217	55 (48 – 62)
Yoshihiro Sato[b]	Endocrinology & Metabolism	JP	26	4	30	119	25 (18 – 34)
Jun Iwamoto[b]	Endocrinology & Metabolism	JP	5	19	24	89	27 (18 – 37)
Kei Satoh	Immunology	JP	0	14	14	28	50 (31 – 69)
Scott S. Reuben	Anesthesiology	US	14	0	14	17	82 (57 – 96)
Zatollah Asemi	Nutrition & Dietetics	IR	3	9	12	13	92 (64 – 100)
Mohammad Reza Safarinejad	Urology & Nephrology	IR	10	1	11	17	65 (38 – 86)
Giuseppe Derosa	Cardiovascular System & Hematology	IT	10	0	10	10	100 (69 – 100)
Hironobu Ueshima[b]	Anesthesiology	JP	3	1	4	120	3 (0.9 – 8)
Hideo Matsumoto	Orthopedics	JP	0	4	4	26	15 (4 – 35)
H.J. Eysenck	Social Psychology	UK	0	1	1	20	5 (0.1 – 25)
Piero Anversa	Cardiovascular System & Hematology	US	0	1	1	19	5 (0.1 – 26)
Annarosa Leri	Cardiovascular System & Hematology	US	0	1	1	19	5 (0.1 – 26)
Jan Kajstura	Cardiovascular System & Hematology	US	0	1	1	18	5 (0.1 – 27)
Walid Kamal Abdelbasset	Food Science	EG	1	0	1	17	6 (0.1 – 29)
Mitsuhiro Osame	Neurology & Neurosurgery	JP	0	1	1	14	7 (0.1 – 34)
Bruno Vellas	Neurology & Neurosurgery	FR	0	1	1	10	1 (0.2 – 45)

Note.

© 2026 Lyu C et al. *JAMA Network Open*.

CI, confidence interval; RCT, randomized controlled trials, DE, Germany; EG, Egypt; FR, France; IT, Italy; IR, Iran; JP, Japan; UK, United Kingdom; US, United States

[a] According to the updated science-wide author databases of standardized citation indexes

[b] Also included on the Retraction Watch leaderboard

© 2026 Lyu C et al. *JAMA Network Open*.

eTable 2. Retracted Randomized Trials Authored by 2024 Top-Cited Scientists With at Least 10 Retractions

Top-cited (single year-long) & ≥ 10 retractions	Subfield[a]	Country	RCTs as 1st author	RCTs as co-author	Total retracted RCTs	Total retracted articles[a]	% retracted RCTs vs articles (95% CI)
Kei Satoh	Immunology	JP	0	14	14	28	50 (31 – 69)
Zatollah Asemi	Nutrition & Dietetics	IR	3	9	12	13	92 (64 – 100)
Mohammad Reza Safarinejad	Urology & Nephrology	IR	10	1	11	17	65 (38 – 86)
Giuseppe Derosa	Cardiovascular System & Hematology	IT	10	0	10	10	100 (69 – 100)
H.J. Eysenck	Social Psychology	UK	0	1	1	20	5 (0.1 – 25)
Walid Kamal Abdelbasset	Food Science	EG	1	0	1	17	6 (0.1 – 29)
Bruno Vellas	Neurology & Neurosurgery	FR	0	1	1	10	1 (0.2 – 45)

Note.
CI, confidence interval; RCT, randomized controlled trials; EG, Egypt; FR, France; IT, Italy; IR, Iran; JP, Japan; UK, United Kingdom
[a] According to the updated science-wide author databases of standardized citation indexes

eTable 3. Correlation Between the Main Variables Extracted

Variable R (P-value)	1	2	3	4	5	6	7
1. Super-retractor (y/n)	1.00	0.861[b] (<0.001)	0.03 (0.99)	-0.757[b] (<0.001)	-0.367[b] (<0.001)	0.724[b] (<0.001)	0.030 (0.99)
2. Top-cited career-long ≥ 10 retractions (y/n)		1.00	0.346[b] (<0.001)	-0.729[b] (<0.001)	-0.403[b] (<0.001)	0.659[b] (<0.001)	0.07 (0.21)
3. Top-cited in 2024 ≥ 10 retractions (y/n)			1.00	-0.082 (0.058)	-0.019 (>0.99)	0.092[b] (0.016)	0.055 (0.63)
4. Publication Year				1.00	0.653[b] (<0.001)	-0.835[b] (<0.001)	-0.144[b] (<0.001)
5. Retraction Year					1.00	-0.132[b] (<0.001)	-0.128\[b] (<0.001)
6. Time to Retraction (days)						1.00	0.097[b] (0.009)
7. Citations (Scopus)[a]							1.00

Note. Values shown are Pearson correlation coefficients.

[a] N=1318

[b] Statistically significant after Sidak correction for multiple comparisons (adjusted α = 0.0024 for 21 comparisons).

© 2026 Lyu C et al. *JAMA Network Open*.

eTable 4. Multivariable Linear Regression Analysis With Total Citations (Scopus) as Dependent Variable

Model	Variable	Coefficient (SE)	*P*-value	Overall *P*-value	R^2
1	Intercept	7.13 (4.52)	0.115	<.001	0.03
	Time to retraction (days)	0.02 (0.003)	<.001		
	Super-retractor present (versus not)[a]	24.7 (14.93)	0.098		
	Time to retraction * Super-retractor interaction	-0.02 (0.004)	<.001		
2	Intercept	10.01 (4.48)	0.026	<.001	0.03
	Time to retraction (days)	0.01 (0.002)	<.001		
	Top-cited career-long ≥ 10 retractions present (versus not)[b]	35.26 (12.98)	0.007		
	Time to retraction * Top-cited career-long ≥ 10 retractions interaction	-0.01 (0.003)	<.001		
3	Intercept	17.14	<.001	0.002	0.01
	Time to retraction (days)	0.004	0.001		
	Top-cited 2024 ≥ 10 retractions present (versus not)[c]	18.71	0.524		
	Time to retraction *Top-cited 2024 ≥ 10 retractions interaction	0.003	0.701		

Note. Multivariable analysis included 1318 articles for which we could retrieve citation data.
[a] 290 articles with a co-author super-retractor versus 1028 with no such co-author
[b] 327 articles with a co-author top-cited career-long versus 991 with no such co-author
[c] 50 articles with a co-author top-cited single-year versus 1268 with no such co-author

© 2026 Lyu C et al. *JAMA Network Open*.

eResults.

Univariate regression analyses stratified by presence of super-retractors (eFigure 1) indicated that their papers versus those with no super-retractors had more baseline citations (intercept (SE)= 31.83 (4.70) versus 7.13 (5.06)), but a slower citation rate over time (slope= 0.0002 citations/day, *P* =0.807 vs 0.02 citations/day, *P* < 0.001). Univariate regression analyses (eFigure 2) similarly indicated that papers with top-cited career-long scientists co-authors versus those without them had more citations at baseline (intercept (SE)= 45.27 (7.93) versus 10.01 (4.89)), but average citation counts remained stable over time (slope= -0.001 citations/day, *P*=0.468 vs 0.01 citations/day, *P* < 0.001).

© 2026 Lyu C et al. *JAMA Network Open*.

eFigure 1. Mean Citations (±95% Confidence Interval) by Survival Time and Superretractor Status in 200-Day Bins (≥5 Papers)

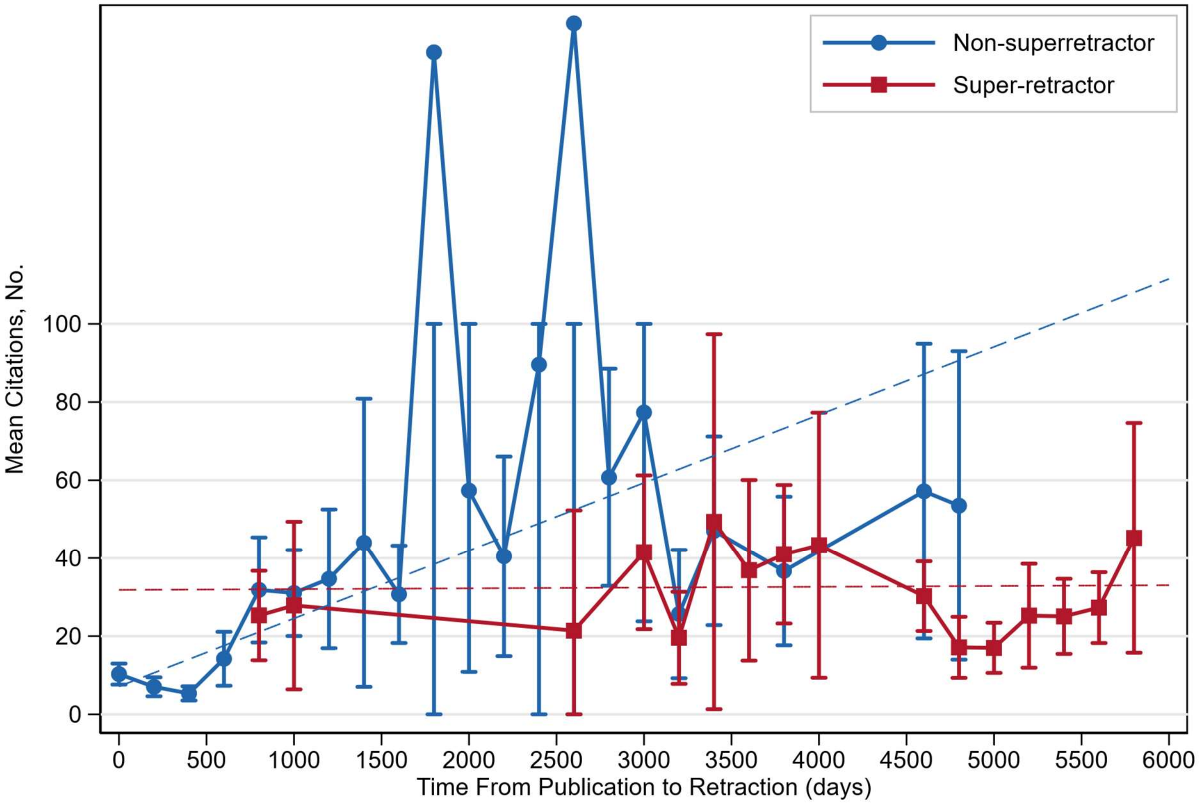

Dashed lines show model predictions. Papers not co-authored by super-retractors (blue circles) showed citation growth (0.017/day, P<.001); super-retractor papers (red squares) did not (0.0002/day, P=.81).
Data restricted to papers with a time-lag of ≤6,000 days between publication and retraction (97 papers) and bins with ≥5 papers (64 papers), leading to 1157 of 1318 papers (88%) plotted.
Confidence intervals capped at 100 for display.

eFigure 2. Mean Citations (±95% Confidence Interval) by Survival Time and Top-Cited Career-Long Status in 200-Day Bins (≥5 Papers)

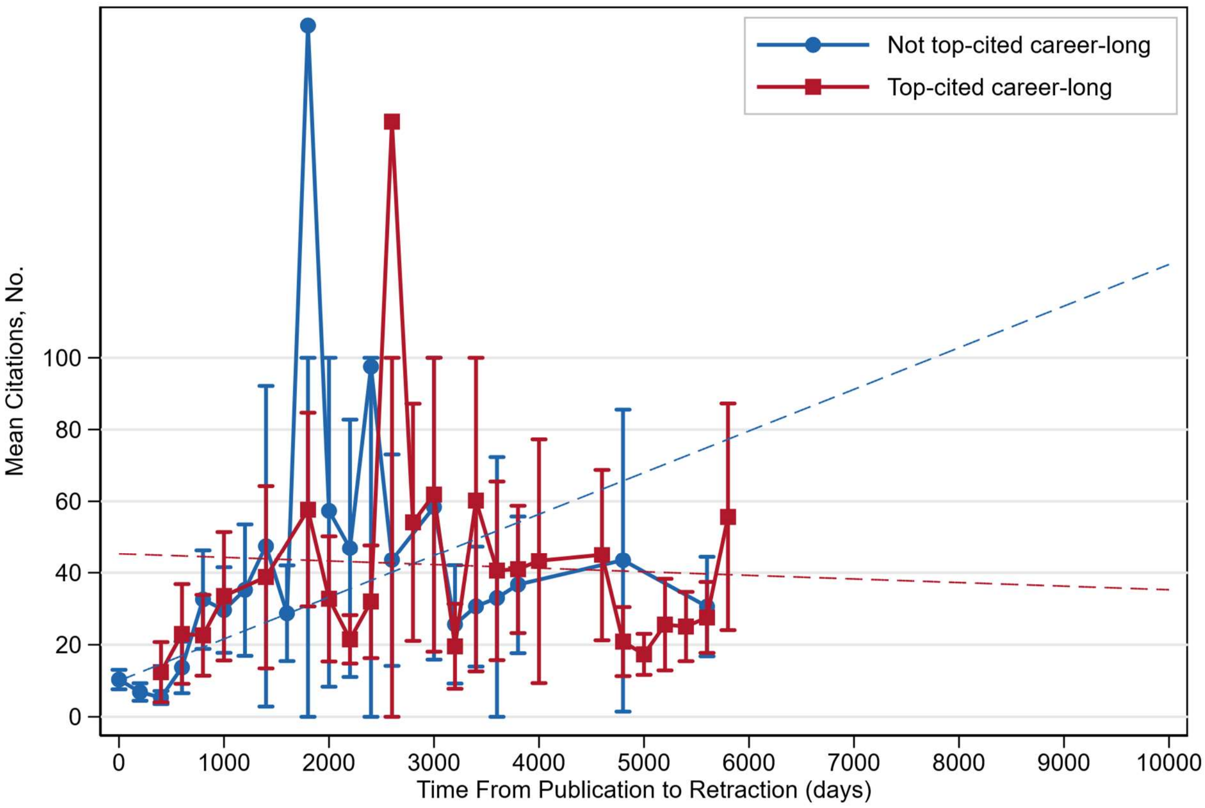

Dashed lines show model predictions. Papers not co-authored by top-cited career-long scientists (blue circles) showed citation growth (0.012/day, P<.001); papers by top-cited career-long authors (red squares) did not (-0.001/day, P=.47).
Data restricted to papers with a time-lag of ≤10000 days between publication and retraction (32 papers) and bins with ≥5 papers (104 papers), leading to 1182 of 1318 papers (90%) plotted.
Confidence intervals capped at 100 for display.

© 2026 Lyu C et al. *JAMA Network Open*.
