# Supplementary material for: Retracted Randomized Clinical Trials From Superretractors and Top-Cited Scientists With Multiple Retractions
Source: JAMA Netw Open. 2026 Apr 15;9(4):e267424. doi: 10.1001/jamanetworkopen.2026.7424 (PMC13084457; doi:10.1001/jamanetworkopen.2026.7424)
Supplement: Supplement 2. — Data Sharing Statement [file jamanetwopen-e267424-s002.pdf]

Data Sharing Statement

Lyu. Retracted Randomized Clinical Trials From Superretractors and Top-Cited Scientists With Multiple Retractions. *JAMA Netw Open*. Published April 15, 2026.
doi:10.1001/jamanetworkopen.2026.7424

Data
Data available: Yes
Data types: Data (not involving human participants)
How to access data: All data is publicly available on Zenodo after publication (10.5281/zenodo.18630545)
When available: With publication

Supporting Documents
Document types: Statistical/analytic code
How to access documents: Statistical code is also be publicly available on Zenodo (https://doi.org/10.5281/zenodo.18630545).
When available: With publication

Additional Information
Who can access the data: Data are be openly available to anyone.
Types of analyses: For any analysis
Mechanisms of data availability: Direct download from Zenodo
